# The prognostic significance of LTBP2 for malignant tumors: Evidence based on 11 observational studies

**DOI:** 10.1097/MD.0000000000029207

**Published:** 2022-05-06

**Authors:** Jianmeng Zhao, Xiaokang Liu, Ke Cong, Jinzhe Chang, Hongqing Shan, Yuenan Zheng

**Affiliations:** a The Second Department of General Surgery, Guangrao County People's Hospital, Guangrao, China; b Department of Medical Oncology, Guangrao County People's Hospital, Guangrao, China.

**Keywords:** latent transforming growth factor-β-binding protein 2, prognosis, risk factor

## Abstract

**Background & aims:**

At present, increasing reports have shown that latent transforming growth factor-β-binding protein 2 (LTBP2) was associated with the prognosis of many types of cancer. We performed rounded analysis to comprehensively analyze and evaluate the prognostic significance of LTBP2 for patients with malignant tumors.

**Methods:**

We identified relevant studies by searching database including PubMed, Embase, Cochrane Library, and Web of Science. The odds ratio with its 95% confidence interval (CI) was used to assess the correlation between LTBP2 and clinicopathologic features or overall survival of patients with cancer. Hazard ratio with its 95% CI was used to explore the prognostic risk factors. The analysis was performed and assessed using Review Manager 5.2.

**Results:**

A total of 11 studies including 2322 participants were included in this systematic review. Pooled results showed that malignant tissues experienced higher incidence of high LTBP2 expression when compared with adjacent or normal tissues. Patients with high LTBP2 expression experienced significantly lower 1-year, 2-year, 3-year, and 4-year overall survival rate, with the pooled odds ratios being 0.26 (95% CI 0.13–0.53; *P* = .0002), 0.27 (95% CI 0.14–0.50; *P* < .0001), 0.26 (95% CI 0.13–0.53; *P* = .0002), and 0.21 (95% CI 0.06–0.73; *P* = .01) respectively. Univariate analysis showed high LTBP2 expression, tumor node metastasis stage, T stage, and N stage were prognostic factors of patients with tumors. Multivariate analysis indicated high LTBP2 expression was an independent prognostic factor.

**Conclusions:**

The present analysis suggested that LTBP2 may have significant association with survival of patients with cancer. High LTBP2 expression was an independent prognostic factor and indicated poor survival.

## 1 Introduction

It is well known that the prognosis of patients with malignant tumors depends on the recurrence and metastasis of tumors. In addition, it has become increasingly clear that tumor microenvironment related to the recurrence of tumor closely. Extracellular matrix (ECM) is an essential component of the stromal microenvironment, which provides structural support and biochemical and physical signals for normal cell function maintenance.^[1–3]^

Recently, latent transforming growth factor-β-binding protein 2 (LTBP2) which was defined as an ECM protein encoded by the fibrillin/LTBP ECM glycoprotein family, has been reported to be associated with the prognosis of patients with many tumors. Accumulating evidence has strongly implied that LTBP2 was involved in ECM formation and played an important role in cell adhesion and elastic fiber aggregation.^[4]^ In recent years, abundant studies regarded the underlying roles of LTBP2 in various tumors. For example, Han et al,^[5]^ found that LTBP2 protein expression was significantly higher in head and neck squamous cell carcinoma tissues, and was associated with lymph node metastasis and higher tumor node metastasis (TNM) stages, suggesting that LTBP2 served as an independent prognostic biomarker in head and neck squamous cell carcinoma (HNSCC). Moreover, Wang et al,^[6]^ reported that LTBP2 protein levels were significantly elevated in pancreatic ductal adenocarcinoma tissues. High levels of LTBP2 were correlated with poor differentiation and advanced TNM stage and predicted worse overall survival (OS) and disease-free survival. da Costa et al,^[7]^ investigated the serum levels of LTBP2 in a prospective cohort of 115 patients with chronic liver disease from Korea between 1999 and 2001, and found that increased serum LTBP2 was detected in 21 subjects who developed HCC, which improved biomarker-based detection of HBV-related HCC. However, the clinical significance of LTBP2 in different tumors was inconsistent.

Therefore, we designed a meta-analysis based on relevant studies to comprehensively analyze and evaluate the prognostic role of LTBP2 in patients with tumors. Besides, to identify whether the incidence of high LTBP2 expression was influenced by many clinicopathologic characteristics, we also analyzed the association between incidence of high LTBP2 expression and clinicopathologic characteristics of patients with tumors. In addition, we also explored the prognostic risk factors of tumor patients.

## 2 Methods and materials

### 2.1 Including and excluding criteria:

Including criteria:

Prospective and retrospective observational studies; the patients included in studies were pathologically diagnosed with malignant tumors; LTBP2 of carcinoma tissues and adjacent tissues were reported; prognostic indicators such as OS and prognostic risk factors could be obtained.

Excluding criteria:

Non-human researches or trials on animals; articles belong to abstracts, letters, editorials, expert opinions, reviews, case reports, or laboratory studies; patients having other primary tumors or severe disease which may affect their survival; studies without sufficient data for analysis; duplicate articles were excluded.

### 2.2 Search strategy:

We identified relevant studies by searching database including PubMed, Embase, Cochrane Library, and Web of Science to February, 2021. Our searching terms and procedures were as follows: “latent transforming growth factor-β-binding protein 2” OR “LTBP2”; “cancer∗” OR “tumor∗” OR “carcinoma∗” OR “neoplasm∗”; “prognosis” OR “survival” OR “outcome” OR “risk factor.” The retrieval formula was as follows: AND, AND. The databases above were searched with these terms and retrieval formula in English. Two investigators who received normative and unitive training beforehand independently screened the titles and abstracts of each study after duplicate references were excluded. Once potential studies which may meet our including criteria were found, their full texts were obtained for further evaluation.

### 2.3 Quality assessment and data extraction:

Two assessors receiving normative training beforehand independently evaluated the quality of all the included studies using the 9-star Newcastle-Ottawa Scale (NOS).^[8]^ The total NOS scores of each study were displayed in the characteristics table (Table 1). The scores were judged according to the 3 aspects of NOS of evaluation: selection, comparability, and outcome between the case group and control group. A study with a NOS score ≥6 is considered experiencing good quality. In addition, in order to observe the bias of our included studies better, the risk of bias for each studies and the risk of bias across all studies were evaluated and shown with figures generated by RevMan 5.2 software (Version 5.2. Copenhagen).^[9]^

**Table 1:** The characteristics of included observational studies for the present meta-analysis.

Study (author and year)	Tumor type	Sample size	Age (median and range)	Cut-off value	LTBP2 detection	RNA extraction	High/positive LTBP2	Follow-up time	Primary outcomes	NOS score
Chan SH (2011)	ESCC	105	66 (34–88)	>25%^∗^	IHC	RT-PCR	35%	6 weeks	The tumor-suppressive role of LTBP-2, survival	8
Chen J (2019)	HCC	60	63.5 (27–87)	>1^†^	IHC	qRT-PCR	68.3%	NR	The relationship between LTBP2 and clinical characteristics, survival	6
da Costa AN (2015)	HCC	684	67	27 ng/mL	IHC	qRT-PCR	NR	NR	LTBP2 expression	7
Gu CJ (2018)	Breast cancer	125	60 (25–89)	Score ≥7^‡^	IHC	PCR	55.2%	>5 years	Prognostic risk factors, OS	6
Han L (2016)	HNSCC	459	57		IHC	qPCR	53.4%		Prognostic risk factors, OS	7
Huang Y (2019)	CRC	483	52 (32–73)	Score ≥7^‡^	IHC	qRT-PCR	28.4%	>5 years	Prognostic risk factors, OS	7
Ren Y (2015)	CAC	59	51.2 (47–68)	Score 1^§^	IHC	qRT-PCR	51%	NR	Prognostic risk factors, OS	7
Turtoi A (2011)	PDAC	62	61 (45–78)	NR	IHC	qRT-PCR	30%	NR	Clinical value for diagnostic and therapeutic applications	7
Wan F (2017)	TC	NR	NR	NR	IHC	RT-qPCR	NR	NR	LTBP2 expression, OS	7
Wang C (2017)	PDAC	111	55 (38–79)	IHC scores ≥4^‡^	IHC	RT-qPCR	55%	>5 years	Correlation and diagnosis of LTBP2, OS, DFS	6
Wang J (2018)	GC	174	54 (44–78)	IHC scores ≥ 6^‡^	IHC	RT-qPCR	54.6%	>5 years	LTBP2 expression, OS	6

CAC = cervical adenocarcinoma, CRC = colorectal cancer, DFS = disease-free survival, ESCC = esophageal cancer, GC = gastric cancer, HCC = hepatocellular carcinoma, HNSCC = head and neck squamous cell carcinoma, IHC = immunohistochemistry, LTBP = latent transforming growth factor-β-binding protein, NR = no report, OS = overall survival, PDAC = pancreas ductal adenocarcinoma, qRT-PCR = quantitative real-time polymerase chain reaction, RT-qPCR = reverse transcription-quantitative polymerase chain reaction, TC = thyroid carcinoma.

∗ Presented as percentage of tumor cells positively stained.

† The relative expression level of LTBP2mRNA in HCC tissues.

‡ The product of the expression intensity of positive cells and the percentage of positive cells.

§ The ratio and intensity of positive-staining cells.

Two same reviewers above extracted the data for analysis based on their intensive reading of included articles, and disagreement was resolved by their discussion. In addition, the other contents included study published year, sample size, tumor type, incidence of high LTBP2 expression, LTBP2 detection, LTBP2 RNA extraction, primary outcome, follow-up time, cut-off value and age (median and range) were also extracted using a standardized form (Table 1). Data collected were input into RevMan 5.2 software for analysis.^[9]^

### 2.4 Statistical analysis

In this meta-analysis, the odds ratio (OR) with its 95% confidence interval (CI) was used to assess the correlation between LTBP2 and clinicopathologic features or OS of patients with cancer. Hazard ratio (HR) with its 95% CI was used to explore the prognostic risk factors. The heterogeneity between studies was evaluated by the chi-square-based *Q* statistical test.^[10]^*P* *≤* .10 was deemed to represent significant heterogeneity, and pooled RR was estimated using a random-effect model (the DerSimonian and Laird method).^[11]^ On the contrary, if statistical study heterogeneity was not observed (*P* > .10), a fixed effects model (the Mantel–Haenszel method)^[12]^ was used. The effects of LTBP2 on survival were considered to be statistically significant if OR 95% CI did not overlap with 1.

Besides, to identify whether the expression of LTBP2 in patients with tumors was influenced by many clinicopathologic characteristics, we also analyzed the association between incidence of high LTBP2 expression and 8 clinicopathologic characteristics including 15 comparing subgroups of patients.

This systematic review and meta-analysis followed the Preferred Reporting Items for Systematic Reviews and Meta-Analyses guidelines^[13]^ and has been assessed in line with assessing the methodological quality of systematic reviews guidelines.^[14]^ The present study was approved by the Ethics Committee of Guangrao County People's Hospital.

## 3 Results

### 3.1 Retrieval results of literature and study characteristics:

After the duplicate studies were removed from the primary retrieval results of 101 studies, 89 studies were initially obtained for potential including by screening the titles and abstracts. Fourty six studies were excluded according to their titles and abstracts and 43 full-texts were obtained for further assessment. After 32 full articles were excluded further, eventually a total of 11 studies including 2322 participants were included in qualitative synthesis.^[5–7,15–22]^ In our included studies, the tumor types referred to esophageal cancer, head and neck squamous cell carcinoma, pancreas ductal adenocarcinoma, cervical adenocarcinoma, thyroid carcinoma, gastric cancer, colorectal cancer, hepatocellular carcinoma and breast cancer. All studies detected LTBP2 using immunohistochemistry method and extracted LTBP2 RNA polymerase chain reaction technique.

**Figure 1:**
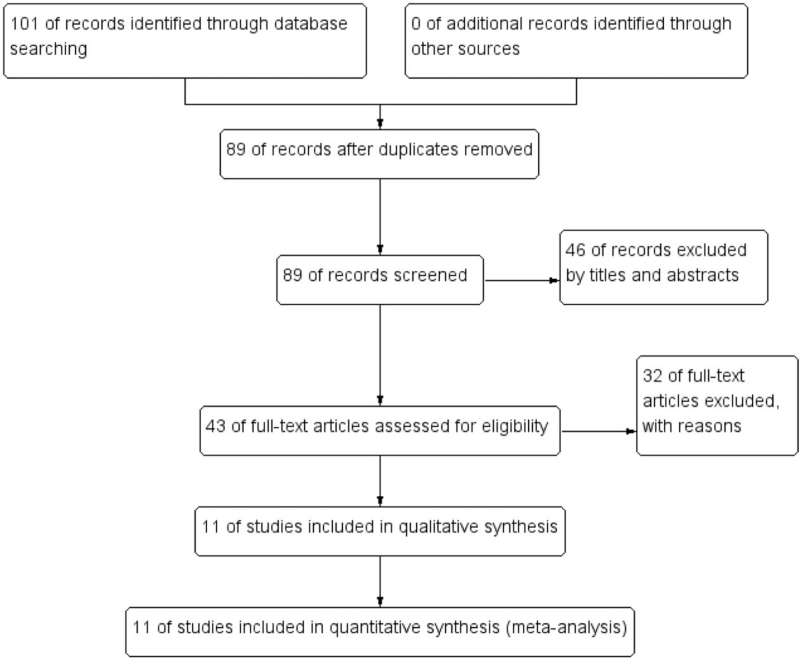
Flow diagram of literature search and selection of included studies for meta-analysis.

The detail search process and summary of studies were showed in study flow diagram (Fig. 1). The other study characteristics of each study were shown in Table 1.

### 3.2 Quality assessment

As Table 1 shows, there is 1 study with a NOS score of 8, 6 studies with a NOS score of 7, 4 studies with a NOS score of 6. According to our definition for good quality, all studies experienced good quality.

**Figure 2:**
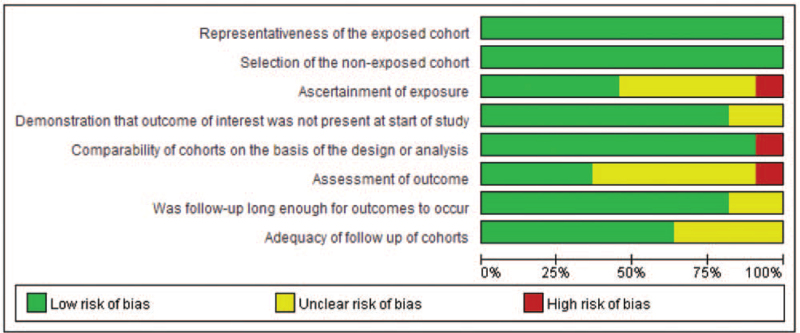
Risk of bias graph: review authors’ judgments about each risk of bias item presented as percentages across all included studies.

**Figure 3:**
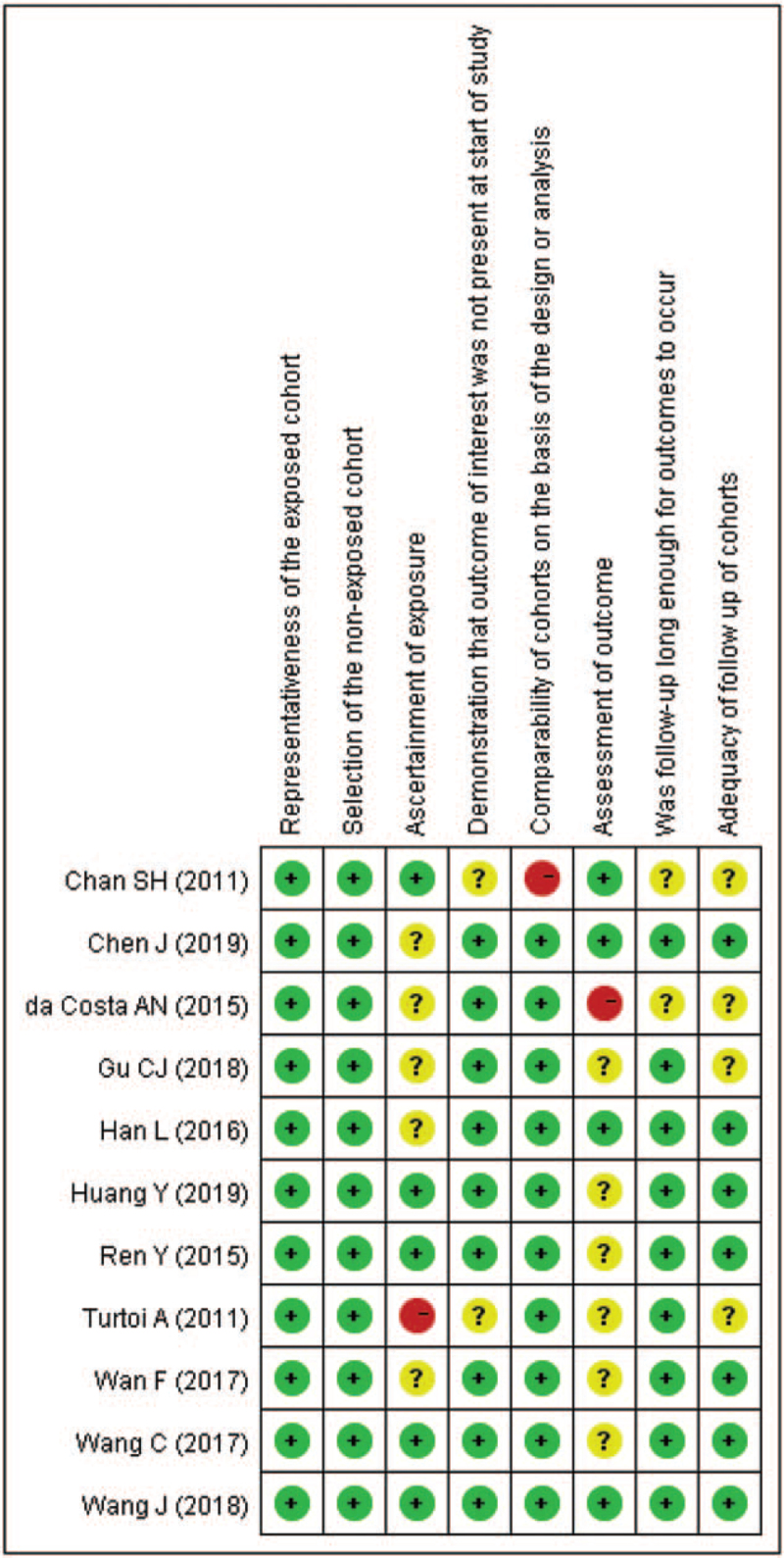
Risk of bias summary: review authors’ judgments about each risk of bias item for each included study.

In addition, to identify the risk of bias of our including studies further, we generated risk of bias graphs. The risk of bias for each study was presented as percentages across all included studies in Fig. 2, and the risk of bias item for each included study is displayed in Fig. 3. The risk of bias graphs indicated generally good methodological quality. In these included studies, they experienced good quality in “section,” including items of representativeness of the exposed cohort and selection of the non-exposed cohort. Besides, comparability issue was low risk of bias in these studies. High risk of bias was observed in “ascertainment of exposure,” “comparability,” and “assessment of outcomes.” Unclear risks of bias were mainly observed in “ascertainment of exposure,” “assessment of outcomes,” and “adequacy of follow-up of cohorts.”

### 3.3 LTBP2 expression in carcinoma and adjacent tissues

Of the included studies for estimating the association between LTBP2 expression and survival of patients with tumors, 7 studies provided 10 sets of comparable data for comparing the LTBP2 expression between carcinoma and adjacent tissues.

**Figure 4:**
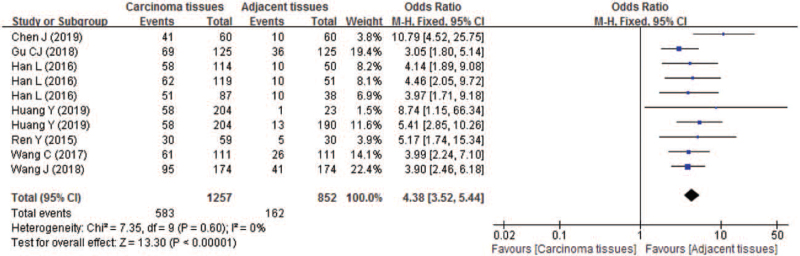
Forest plot of comparison between carcinoma and adjacent tissues regarding to the incidence of high LTBP2 expression. LTBP2 = latent transforming growth factor-β-binding protein 2.

Our overall analysis results showed that, compared with normal or adjacent tissues, the incidence of high LTBP2 expression was more than 4 folds in carcinoma tissues, with the pooled OR being 4.38 (95% CI 3.52–5.44; *P* < .00001) (Fig. 4). The pooled analysis was estimated using fixed-effect model because no significant heterogeneity (*P* = .60 and *I*^2^ = 0%) between studies was found.

### 3.4 The prognostic effect of LTBP2 expression on OS

**Figure 5:**
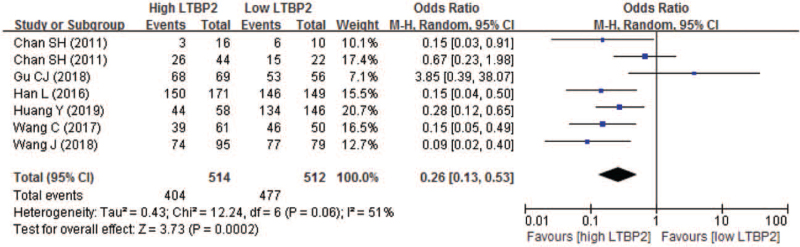
Forest plot of comparison between high and low LTBP2 expression regarding to 1-year overall survival. LTBP2 = latent transforming growth factor-β-binding protein 2.

Of the included studies, 6 studies with 7 sets of comparable data compared the 1-year OS between high and low LTBP2 expression. The 1-year OS rate in high and low LTBP2 expression was 78.6% and 93.2% respectively. Our result showed that patients having high LTBP2 expression experienced worse 1-year OS, with the pooled OR being 0.26 (95% CI 0.13–0.53; *P* = .0002) (Fig. 5).

**Figure 6:**
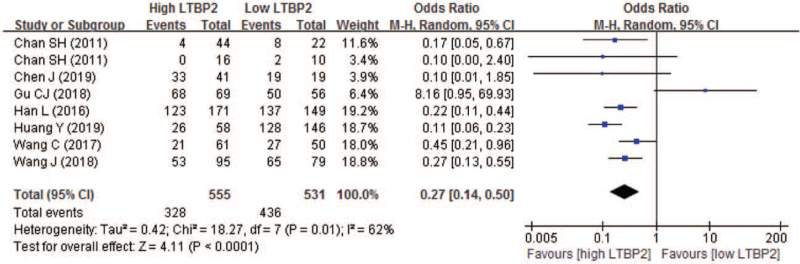
Forest plot of comparison between high and low LTBP2 expression regarding to 2-year overall survival. LTBP2 = latent transforming growth factor-β-binding protein 2.

Seven studies with 8 sets of comparable data compared the 2-year OS between high and low LTBP2 expression. The 2-year OS rate in high and low LTBP2 expression was 59.1% and 82.1% respectively. Pooled result indicated patients having high LTBP2 expression also experienced significantly poor 2-year OS, with a pooled OR of 0.27 (95% CI 0.14–0.50; *P* < .0001) (Fig. 6).

**Figure 7:**
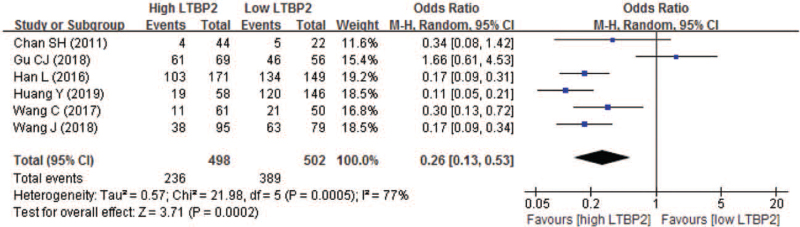
Forest plot of comparison between high and low LTBP2 expression regarding to 3-year overall survival. LTBP2 = latent transforming growth factor-β-binding protein 2.

Six studies compared the 3-year OS between high and low LTBP2 expression. The 3-year OS rate in high and low LTBP2 expression patients was 60.7% and 77.5% respectively. Pooled result indicated patients having high LTBP2 expression also experienced significantly poor 3-year OS, with a pooled OR of 0.26 (95% CI 0.13–0.53; *P* = .0002) (Fig. 7).

**Figure 8:**
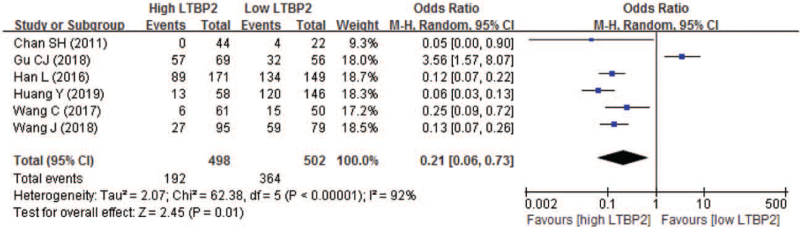
Forest plot of comparison between high and low LTBP2 expression regarding to 4-year overall survival. LTBP2 = latent transforming growth factor-β-binding protein 2.

There also are 6 studies comparing the 4-year OS between high and low LTBP2 expression. The 4-year OS rate in high and low LTBP2 expression patients was 38.6% and 72.5% respectively. Pooled result also indicated that patients having high LTBP2 expression also experienced significantly lower 4-year OS, with a pooled OR of 0.21 (95% CI 0.06–0.73; *P* = .0002) (Fig. 8).

Because of significant heterogeneity between studies, random effect models were used for above combined analyses.

### 3.5 The association between clinicopathologic features and LTBP2 expression

In order to identify whether the LTBP2 expression was influenced by many of the clinicopathologic characteristics of patients with tumors, we analyzed the association between high LTBP2 expression and 8 clinicopathologic characteristics including 15 comparing subgroups (Table 2). The clinicopathologic characteristics items included age, gender, tumor grade, tumor size, N stage, T stage, M stage, and TNM stage. Our analysis results showed that significant associations were found in N stage, T stage, M stage, and TNM stage (Table 2). In N stage, compared with N1 and N2, patients with N0 experienced lower incidence of high LTBP2 expression (OR 0.34; 95% CI 0.19–0.60; *P* = .0002 and OR 0.14; 95% CI 0.05–0.42; *P* = .0005 respectively). Compared with T3/4, patients with T1/2 experienced lower incidence of high LTBP2 expression, with the pooled OR being 0.38 (95% CI 0.21–0.69; *P* = .001). Compared to M1, patients with M0 experienced lower incidence of high LTBP2 expression, with the pooled OR being 0.16 (95% CI 0.07–0.35; *P* < .0001). Meanwhile, when compared the incidence of high LTBP2 expression in patients with TNM III, the same result was also found in TNM stage I and II (OR 0.11; 95% CI 0.04–0.33; *P* < .0001 and OR 0.46; 95% CI 0.23–0.92; *P* = .03 respectively). No significant association between high LTBP2 expression and age, gender, tumor grade, and tumor size was observed (Table 2).

**Table 2:** The comparison between LTBP2 expression and clinicopathologic features.

		Pooled results			Heterogeneity		
Subgroups	Number of pts	OR	95% CI	*P* value	*I* ^2^	*P*_*h*_ value	Analytical effect model
Age, yr	993	0.85	0.66, 1.11	.24	0%	.72	Fixed-effect model
Gender	809	1.32	0.98, 1.78	.07	30%	.23	Fixed-effect model
Tumor grade							
Well vs moderate	331	0.81	0.52, 1.27	.36	0%	.78	Fixed-effect model
Well vs poor	340	0.89	0.24, 3.27	.86	72%	.03	Radom-effect model
Moderatel vs poor	671	0.72	0.47, 1.09	.12	22%	.28	Fixed-effect model
Tumor size, cm	295	0.61	0.24, 1.52	.29	72%	.03	Radom-effect model
N stage	299	0.34	0.19, 0.60				
N0 vs N1	299	0.34	0.19, 0.60	.0002	0%	.92	Fixed-effect model
N0 vs N2	144	0.14	0.05, 0.42	.0005			
N0 vs N1, 2, 3	957	0.51	0.24, 1.07	.07	83%	<.0001	Radom-effect model
T stage							
T1, 2 vs T3, 4	751	0.38	0.21, 0.69	.001	53%	.10	Radom-effect model
M stage							
M0 vs M1	489	0.16	0.07, 0.35	<.0001	28%	.25	Fixed-effect model
TNM stage							
I vs II	278	0.58	0.14, 2.38	.45	79%	.009	Radom-effect model
I vs III	157	0.11	0.04, 0.33	<.0001	0%	1.0	Fixed-effect model
II vs III	164	0.46	0.23, 0.92	.03	0%	0.71	Fixed-effect model
I+II vs III+IV	725	0.83	0.36, 1.92	.67	77%	.001	Radom-effect model

CI = confidence intervals, LTBP2 = latent transforming growth factor-β-binding protein 2, OR = odds ratio, TNM = tumor node metastasis.

### 3.6 The prognostic risk factors of OS

We explored the prognostic risk factors of OS for patients with tumors. As Table 3 shows, the univariate analysis indicated LTBP2 expression (HR 3.2; 95% CI 2.43–4.23; *P* < .0001), TNM stage (HR 1.80; 95% CI 1.02–3.20; *P* = .04), T stage (HR 1.82; 95% CI 1.04–3.18; *P* = .04), and N stage (HR 2.07; 95% CI 1.28–3.36; *P* = .003) were risk factors of prognosis of patients with tumors. No significant results were observed in gender, age, tumor differentiation, M stage, and tumor size. Multivariate analysis found only LTBP2 expression was the risk factor of OS for patients with tumors.

**Table 3:** The pooled analysis of prognostic factors of overall survival.

		Univariate analysis			Multivariate analysis		
Subgroups	Number of pts	HR	95% CI	*P* value	HR	95% CI	*P* value
LTBP2 expression	758	3.2	2.43, 4.23	<.0001	3.41	2.54, 4.57	<.0001
Gender	1227	1.13	0.89, 1.42	.31			
Age	1352	1.20	0.99, 1.46	.06			
Differentiation	1352	0.81	0.48, 1.37	.44	0.99	0.65, 1.51	.96
TNM stage	1352	1.80	1.02, 3.20	.04	1.13	0.61, 2.06	.70
T stage	1227	1.82	1.04, 3.18	.04	1.36	0.91, 2.03	.14
N stage	1352	2.07	1.28, 3.36	.003	1.23	0.94, 1.62	.14
M stage	768	1.78	0.43, 7.33	.42	1.45	0.87, 2.42	.15
Tumor size	236	1.03	0.73, 1.45	.86			

CI = confidence intervals, HR = hazard ratio, LTBP2 = latent transforming growth factor-β-binding protein 2, TNM = tumor node metastasis.

### 3.7 Publication bias

**Figure 9:**
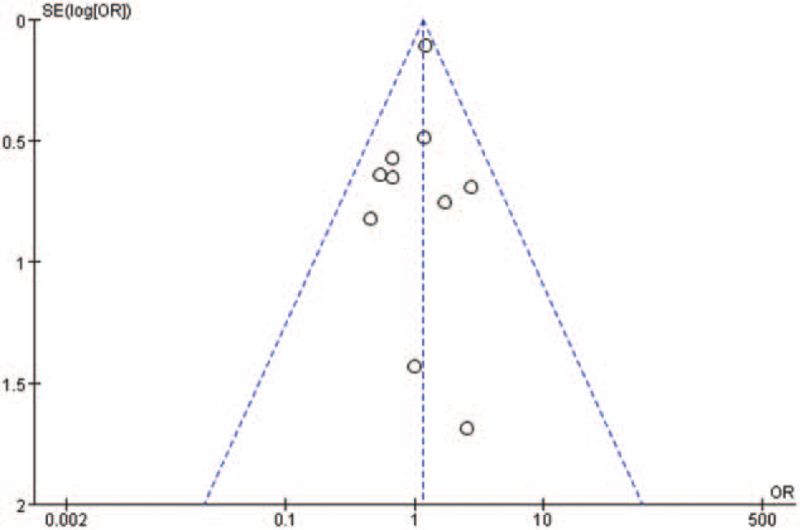
Funnel plot of comparison between high LTBP2 and low LTBP2 in 30-day/in-hospital all-cause mortality.

Begg funnel plot was generated to assess publication bias in the included studies. As shown in Fig. 9, the plots displayed no obvious asymmetry and showed no clear evidence of publication.

## 4 Discussion and conclusion

Recently, increasing interests have been given to the relevance of LTBP2 expression and malignancies. The TGF-β signaling pathway regulates various cellular processes including proliferation, apoptosis, differentiation, ECM modification, cytokine secretion and migration, in normal and malignant cells.^[23,24]^ Nevertheless, TGF-β has contrasting functions in cancer, acting as a tumor suppressor in the development of pre-malignant tumors and as an oncogene in the progression of advanced-stage tumors, particularly during tumor cell invasion and metastasis.^[25,26]^ The majority of cells secrete TGF-β in a latent form, non-covalently bound to TGF-β propeptide (latency-associated peptide [LAP]).^[27]^ LAP associates with LTBPs, which may assist with the proper folding of TGF-β precursor protein, aiding its secretion as well as directing its association with the ECM.^[28]^ LTBP1 promotes TGF-β activation by anchoring to the ECM and creating traction when LAP is bound by cell surface integrins. This traction facilitates LAP deformation and aids in the release of TGF-β from LAP-LTBP complexes, which is essential for the activation of TGF-β.^[29]^ However, LTBP2 is different from other LTBPs as it is the only member that does not bind to latent TGF-β.^[30]^ It has been demonstrated that LTBP1 and LTBP2 exhibited comparable binding affinities for fibrillin-1 because they compete for the same binding site.^[31]^ This led to the hypothesis that LTBP2 may indirectly modulate the TGF-β activation by releasing LTBP1 from microfibrils. However, the definite function of LTBP2 in regulating TGF-β remains unclear, although tumor-promoting and tumor-suppressive functions have been proposed for LTBP2.^[5,32]^

Though the exact effect of LTBP2 on malignant tumors were not clear, many clinical researches have explored its impact on the prognosis of patients with tumors. However, there results remained inconsistency. Thus, we performed this analysis in order to comprehensively analyze and evaluate the prognostic significance of LTBP2 for patients with malignant tumors. Our pooled results indicated that LTBP2 expressed more in carcinoma tissues than normal or adjacent tissues, which could be used as an indicator for malignant tumors. In addition, it was demonstrated that both the early and mid- or long-term OS rate was significantly lower in patients with high LTBP2 expression than low LTBP2 expression. The incidence of high LTBP2 expression was higher in stage III or IV patients than stage I or II. This result was consistent with our conventional cognitive that late stage patients always experienced poor prognosis. Our multivariate analysis results indicated that only LTBP2 expression was an independent prognostic risk factor.

There were several deficiencies for the present meta-analysis. The greatest limitation was the inconsistency of cut-off value of high LTBP2 expression. Chan et al^[15]^ used percentage of tumor cells positively stained >25%, but Chen et al^[16]^ used the relative expression level of LTBP2mRNA in HCC tissues >1 as the cut-off value. The cut-off value of high LTBP2 expression was present as the product of the expression intensity of positive cells and the percentage of positive cells in several studies.^[6,17,18,22]^ The second limitation was the variation of the clinical stages of patients. As is well known, clinical stage, especially TNM stage of tumors, is one of the main factors of tumor patients’ prognosis and is most commonly used as the predictor for prognosis of tumor patients. Because of the heterogeneity and small number of included studies, we failed to perform subgroup analysis according to TNM stage of patients. Meanwhile, the significant association between incidence of high LTBP2 expression and TNM stage was found in our analysis, thus the stage of patients may influence the analysis results of survival results. Another limitation was the difference of tumor types. At present, there was no research exploring and comparing the LTBP2 expression in difference tumor tissues. Thus, this analysis was an initial exploration for the effect of LTBP2 expression on cancer. In the included studies, the incidence of high/positive LTBP2 expression was ranged from 28.4% in colorectal cancer to 68.3% in hepatocellular carcinoma. Thus, the expression of LTBP2 may be different in different tumor tissues, which may lead to any risk of bias of analysis. However, limited to the study number, we failed to perform subgroup analysis according to different cancers. Finally, the prognosis of cancer patients are multifactorial and prognosis of tumor patients are also influenced by multiple other factors, such as age, follow-up time, adjuvant therapy, and tumor size, histological type, and venous involvement, which should also be taken into consideration when designing a research.

Future studies should clear the cut-off value of LTBP2 in different tumor tissues. In addition, because LTBP2 expression was be detected in tumor tissues, further studies should explore the detection of LTBP2 expression in peripheral circulation. More significant, the correlation between LTBP2 expression and prognosis or response to therapy should be explored, which could be used to adjuvant therapy. In addition, future researches should explore and compare the difference of LTBP2 expression in different tumor tissue, which may contribute to clearing the cut-off value of LTBP2 in different tumor tissues.

In conclusion, the present analysis suggested that LTBP2 may have significant association with survival of patients with cancer. Both the early and mid- or long-term OS rate was significantly lower in patients with high LTBP2 expression than low LTBP2 expression. Our pooled results indicated that LTBP2 expressed more in carcinoma tissues than normal or adjacent tissues, which could be used as an indicator for malignant tumors. In addition, it was indicated that high LTBP2 expression was an independent prognostic factor of patients.

## Author contributions

**Conceptualization:** Hongqing Shan, Xiaokang Liu, Yamin Zhang, Yang Li, Yuenan Zheng, Zhe Qiang.

**Data curation:** Xiaokang Liu, Yang Li.

**Formal analysis:** Jiancun Hou, Jianmeng Zhao, Xiaokang Liu, Yang Li.

**Funding acquisition:** Yuenan Zheng, Zhe Qiang.

**Methodology:** Jiancun Hou, Jianmeng Zhao, Xiaokang Liu, Yang Li.

**Software:** Hongqing Shan, Jian Wang, Jiancun Hou, Jianmeng Zhao, Jinzhe Chang, Ke Cong, Qing Tian, Yamin Zhang, Yuenan Zheng, Zhe Qiang.

**Visualization:** Hongqing Shan, Ke Cong, Qing Tian, Yamin Zhang.

**Writing – original draft:** Hongqing Shan, Jian Wang, Jinzhe Chang, Ke Cong, Qing Tian, Xiaokang Liu, Yamin Zhang, Yang Li, Yuenan Zheng, Zhe Qiang.

**Writing – review & editing:** Jian Wang, Jinzhe Chang, Xiaokang Liu, Yang Li, Yuenan Zheng, Zhe Qiang.
